# Complete mitochondrial genome analyses confirm that bat *Polychromophilus* and ungulate *Plasmodium* constitute a distinct clade independent of other *Plasmodium* species

**DOI:** 10.1038/s41598-023-45551-z

**Published:** 2023-11-20

**Authors:** Juthathip Poofery, Thongchai Ngamprasertwong, Duriyang Narapakdeesakul, Apinya Arnuphapprasert, Yudhi Ratna Nugraheni, Suchansa Thanee, Masahito Asada, Osamu Kaneko, Morakot Kaewthamasorn

**Affiliations:** 1 Veterinary Parasitology Research Unit, Department of Pathology, Faculty of Veterinary Science, Chulalongkorn University, Bangkok, Thailand; 2 Department of Biology, Faculty of Science, Chulalongkorn University, Bangkok, Thailand; 3 Veterinary Pathobiology Graduate Program, Faculty of Veterinary Science, Chulalongkorn University, Bangkok, Thailand; 4 The International Graduate Program of Veterinary Science and Technology (VST), Faculty of Veterinary Science, Chulalongkorn University, Bangkok, Thailand; 5 Department of Parasitology, Faculty of Veterinary Medicine, Universitas Gadjah Mada, Sleman, Indonesia; 6 Research Unit for Global Infection Control, National Research Center for Protozoan Diseases, Obihiro University of Agriculture and Veterinary Medicine, Obihiro, 080-8555 Japan; 7 Department of Protozoology, Institute of Tropical Medicine (NEKKEN), Nagasaki University, Nagasaki, 852-8523 Japan

**Keywords:** Molecular evolution, Phylogenetics, Infectious diseases, Ecology, Evolution, Microbiology, Molecular biology, Zoology

## Abstract

In recent phylogenetic studies, bat *Polychromophilus* and ungulate *Plasmodium*, two relatively understudied haemosporidian parasites within the Apicomplexa phylum, have often been overlooked. Instead, the focus has been primarily on haemosporidian parasites in primates, rodents, and birds. Several phylogenetic analyses of bat *Polychromophilus* have relied on limited datasets and short informative DNA sequences. As a result of these inherent limitations, the substantiation of their evolutionary stance has encountered a diminished degree of robust validation. This study successfully obtained complete mitochondrial genome sequences from 11 *Polychromophilus* parasites originating from *Hipposideros gentilis* and *Myotis siligoensis* bats for the first time. Additionally, the authors have sequenced the apicoplast *caseinolytic protease C* genes from *Polychromophilus murinus* and a potentially new *Polychromophilus* species. These mitochondrial genomes range in length from 5994 to 6001 bp and consist of three protein-coding genes (PCGs), seven small subunit ribosomal RNA genes (SSU rRNA), 12 large subunit ribosomal RNA genes (LSU rRNA), and seven miscellaneous RNA genes. Phylogenetic analyses using Bayesian Inference and Maximum Likelihood methods indicated robust support for the grouping of ungulate *Plasmodium* and bat *Polychromophilus* in a single clade separate from other *Plasmodium* spp., confirming previous reports, albeit with stronger evidence in this study. The divergence between *Polychromophilus* in bats and *Plasmodium* in ungulates occurred approximately 29.61 to 55.77 million years ago (Mya), with a node age estimated at 40.63 Mya. These findings highlight that the genus *Plasmodium*, which includes species found in ungulates, birds, reptiles, and other mammals, does not form a monophyletic group. By incorporating *Polychromophilus* in bats and *Plasmodium* in ungulates, this study contributes significantly to understanding the phylogenetic relationships within the Haemosporida order. It provides valuable insights into the evolutionary history and interconnections among these diverse parasites, thereby expanding knowledge in this field.

## Introduction

The order Haemosporida encompasses a diverse group of obligatory intracellular parasites, known as haemosporidians, which infect a wide range of reptiles, birds, and mammals^1,2^. Phylogenetic analyses play a crucial role in unraveling the evolutionary history of these parasite species, shedding light on their ancestral origins and transitions between host groups. To ensure accurate phylogenetic inference and comprehensive evolutionary studies, it is imperative to increase taxon sampling and employ appropriate molecular markers^3–5^. In-depth investigations of mitochondrial genes and genomes of haemosporidian parasites in primates, rodents, reptiles, and birds have been conducted for various purposes, such as species identification, phylogenetic inference, and evolutionary studies^6–9^. Mitochondrial genomes exhibit consistent A + T content, particularly among *Plasmodium* species, mitigating the risk of model misspecification in phylogenetic analyses^9^. Notably, both mitochondrial genome-based species trees and multilocus datasets yielded congruent results across major clades^9,10^.

The mitochondrial genome of haemosporidian parasites, characterized by its compact size, stands as the smallest among eukaryotes^11–13^. Spanning approximately 6 kb, this genome only encodes three proteins associated with the electron transport chain: *cytochrome c oxidase subunit 1* (*cox1*), *cytochrome c oxidase subunit 3* (*cox3*), and *cytochrome b* (*cytb*). Additionally, it exhibits highly fragmented small subunit (SSU) and large subunit (LSU) ribosomal ribonucleic acid (rRNA) genes^14^. Several phylogenetic analyses have unveiled the polyphyly of *Plasmodium* spp. and raised concerns over the taxonomic inconsistencies or inadequate support for the phylogenetic tree within the order Haemosporida^10,15–18^. Moreover, the phylogenetic relationship between ungulate *Plasmodium* parasites and bat *Polychromophilus* within the order Haemosporida remains not completely resolved in several studies, partly due to the limited length of genetic markers employed or the high percentage of missing data. For instance, Martinsen et al.^19^ utilized 1370 bp of the *cytb, cox1, adenylosuccinate lyase* (*asl*), and *caseinolytic protease C* (*clpC*) genes in their study. Templeton et al.^16^ employed *cytb, cox1,* and *clpC* gene sequences, encompassing 2794 bp (excluding gaps), to investigate 53 taxa. Galen et al.^10^ analyzed a dataset of 21 genetic markers spanning 19,851 bp across 58 ingroup taxa, though the dataset contained over 30% of missing data. Unfortunately, *Polychromophilus* was not included in the dataset used by Pacheco et al.^20^, who examined a dataset of 108 sequences, encompassing 5108 bp (excluding gaps), derived from mitochondrial genome sequences.

To date, a total of 1469 bat species (Order: Chiroptera) worldwide have been recognized officially^21^. Bats possess lightweight physiology to enable flight, resulting in relatively modest blood volume compared to other mammals. For instance, the Kitti's hog-nosed bat weighs less than a penny. Furthermore, bats are strictly protected animals in several countries including Thailand, leading to the exclusion of bat haemosporidian parasites from previous evolutionary studies. Among the haemosporidian parasites identified in bats, the genera *Plasmodium, Hepatocystis, Nycteria*, and *Polychromophilus* are well-recognized and receive substantial scientific interest. While *Plasmodium* and *Hepatocystis* infect a diverse range of mammals, *Nycteria* and *Polychromophilus* have been found in bats exclusively^22^. *Polychromophilus* parasites have been discovered in bats belonging to the families Miniopteridae, Vespertilionidae, Emballonuridae, Hipposideridae, Rhinolophidae and Rhinonycteridae ^22–28^. Specifically, *Polychromophilus corradetti* and *Polychromophilus adami* were isolated from *Miniopterus* spp. bats in Central Africa (Gabon and Congo)^29^, while *Polychromophilus deanei* was identified in *Myotis nigricans* bats (Vespertilionidae) in the Amazon region of Brazil^30^. The descriptions for *Po. corradetti, Po. adami,* and *Po. deanei* were based solely on their morphological characteristics, and no DNA evidence has been deposited in public databases thus far. Although the complete mitochondrial genome sequence of *Nycteria* parasites has been investigated in a previous study^31^, the mitochondrial genome of *Polychromophilus* remains incompletely characterized. In this study, the authors aimed to elucidate the phylogenetic position of *Polychromophilus* parasites concerning ungulate malaria parasites by obtaining the complete mitochondrial genome sequences of *Polychromophilus* sp. parasites isolated in Thailand. Additionally, this study reports on the discovery of a potentially new *Polychromophilus* species found in *Hipposideros gentilis* bats.

## Results

### Features of the mitochondrial genomes of *Polychromophilus*

**Figure 1:**
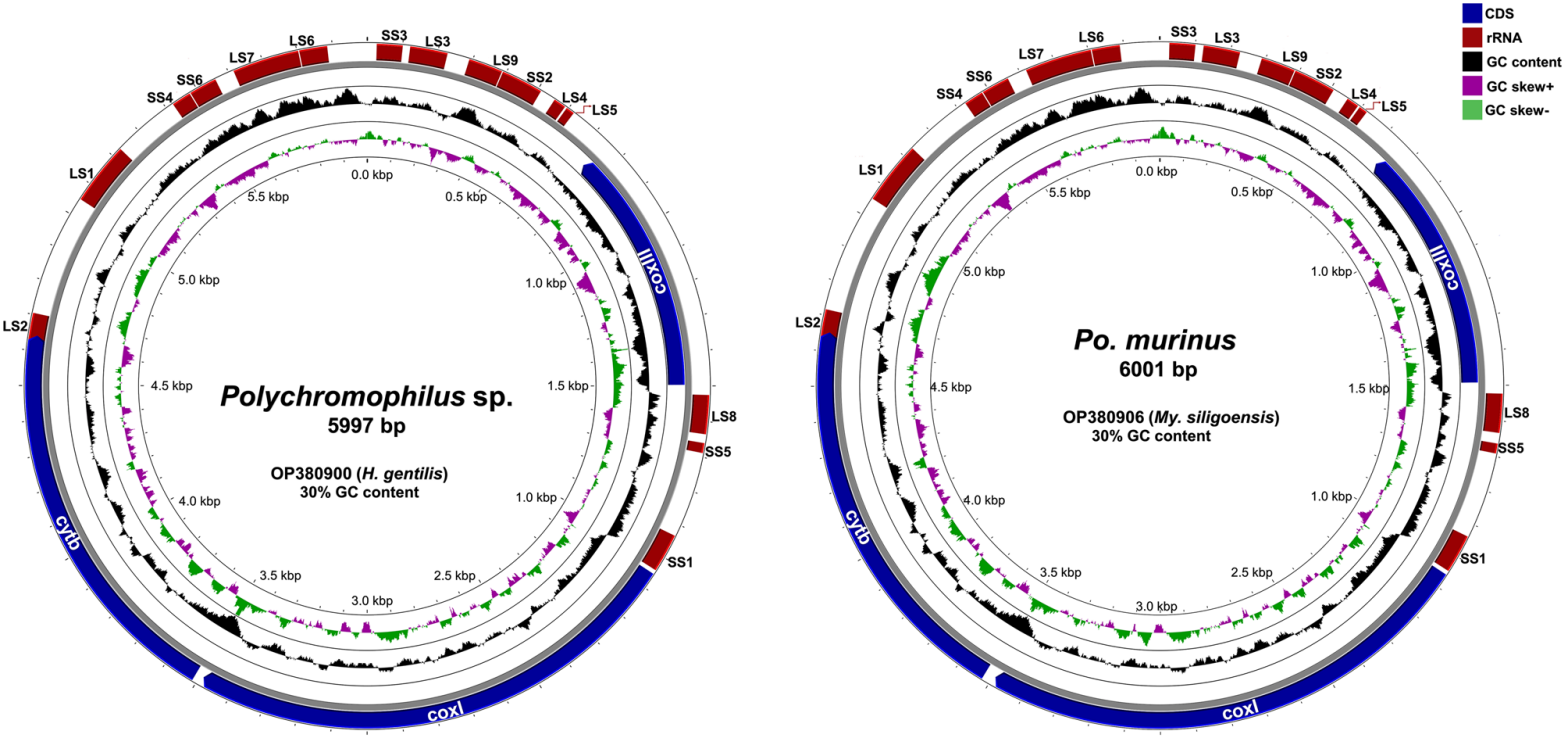
Mitochondrial genome maps of *Polychromophilus* sp. (left) and *Po. murinus* (right). The inner circles are color-coded to represent GC-skew (−) in green and GC-skew ( +) in violet. The middle circles illustrate the GC content. The outer circles depict the organization of the three protein-coding genes (PCGs) in dark blue and the rRNA genes in brown.

*Polychromophilus* sp. infections were detected in two individuals of the bat species *Hipposideros gentilis* (sample IDs THBat19_19 and THBat20_171), while *Polychromophilus murinus* infections were verified from two individuals of the bat species *Myotis siligorensis* (sample IDs THBat_20_229 and THBat20_242). As an aggregate outcome, we successfully acquired a total of 11 complete mitochondrial genome sequences from *Polychromophilus* parasites derived from four bats, as summarized in Table 1. The genetic divergence between these haplotypes ranged from 0.001 to 0.005 in sample ID THBat20_171, 0.006 in sample ID THBat20_229, and from 0.003 to 0.010 in sample ID THBat20_242 (Table S1). Among these sequences, *Po. murinus* displayed genome sizes of 5994 bp (2 sequences from THBat20_229) and 6001 bp (4 sequences from THBat20_242), while *Po*. sp. exhibited genome sizes of 5997 bp (4 sequences from THBat20_171) and 5998 bp (1 sequence from THBat19_191). These genomes encompassed three protein-coding genes (PCGs), seven small subunit ribosomal RNA genes (SSU rRNA), 12 large subunit ribosomal RNA genes (LSU rRNA), and seven miscellaneous RNA genes, as depicted in Fig. 1 and Tables S2 and S3. BLASTN similarity searches and sequence annotations confirmed that the mitochondrial genomes of *Polychromophilus* shared the same organizational pattern of PCGs and highly fragmented rRNAs observed in *Plasmodium* species. The initiation codons ATG were identified for the *cytb, cox1*, and *cox3* genes. Specifically, the *cytb* and *cox1* genes were found in frame 1 of the plus strand, while the *cox3* gene was in frame 2 of the negative strand. The protein-coding regions were terminated by stop codons, with TAA identified for *cytb* and *cox3*, and TAG for *cox1*.

**Table 1:** Bat host species, mitochondrial genome size, and gene coverage of *cox3, cox1*, and *cytb* genes in *Polychromophilus* sp. and *Po. murinus* obtained from the current study.

Sample ID	Host species	Parasite	Accession no.	Mitochondrial genome size (bp)	Protein coding sequence spanning		
					*cox3* (start–stop)	*cox1* (start–stop)	*Cytb* (start–stop)
THBat19_191_C1	*H. gentilis*	*Polychromophilus* sp.	OP380899	5998	1493–741	2056–3489	3514–4644
THBat20_171_C1	*H. gentilis*	*Polychromophilus* sp.	OP380900	5997	1493–741	2055–3488	3513–4643
THBat20_171_C2	*H. gentilis*	*Polychromophilus* sp.	OP380901	5997	1493–741	2055–3488	3513–4643
THBat20_171_C3	*H. gentilis*	*Polychromophilus* sp.	OP380902	5997	1493–741	2055–3488	3513–4643
THBat20_171_C4	*H. gentilis*	*Polychromophilus* sp.	OP380903	5997	1493–741	2055–3488	3513–4643
THBat20_229_C1	*My. siligoensis*	*Po. murinus*	OP380904	5994	1491–739	2059–3492	3517–4647
THBat20_229_C2	*My. siligoensis*	*Po. murinus*	OP380905	5994	1491–739	2059–3492	3517–4647
THBat20_242_C1	*My. siligoensis*	*Po. murinus*	OP380906	6001	1491–739	2059–3492	3524–4654
THBat20_242_C2	*My. siligoensis*	*Po. murinus*	OP380907	6001	1491–739	2059–3492	3524–4654
THBat20_242_C3	*My. siligoensis*	*Po. murinus*	OP380908	6001	1491–739	2059–3492	3524–4654
THBat20_242_C4	*My. siligoensis*	*Po. murinus*	OP380909	6001	1491–739	2059–3492	3524–4654

The numerical component within each sample ID signifies the year of sample collection, the specific bat identification, and the corresponding clone number.

### Phylogenetic relationships of *Polychromophilus *with other haemosporidian parasites based on whole mitochondrial genome and *clpC* sequences

**Figure 2:**
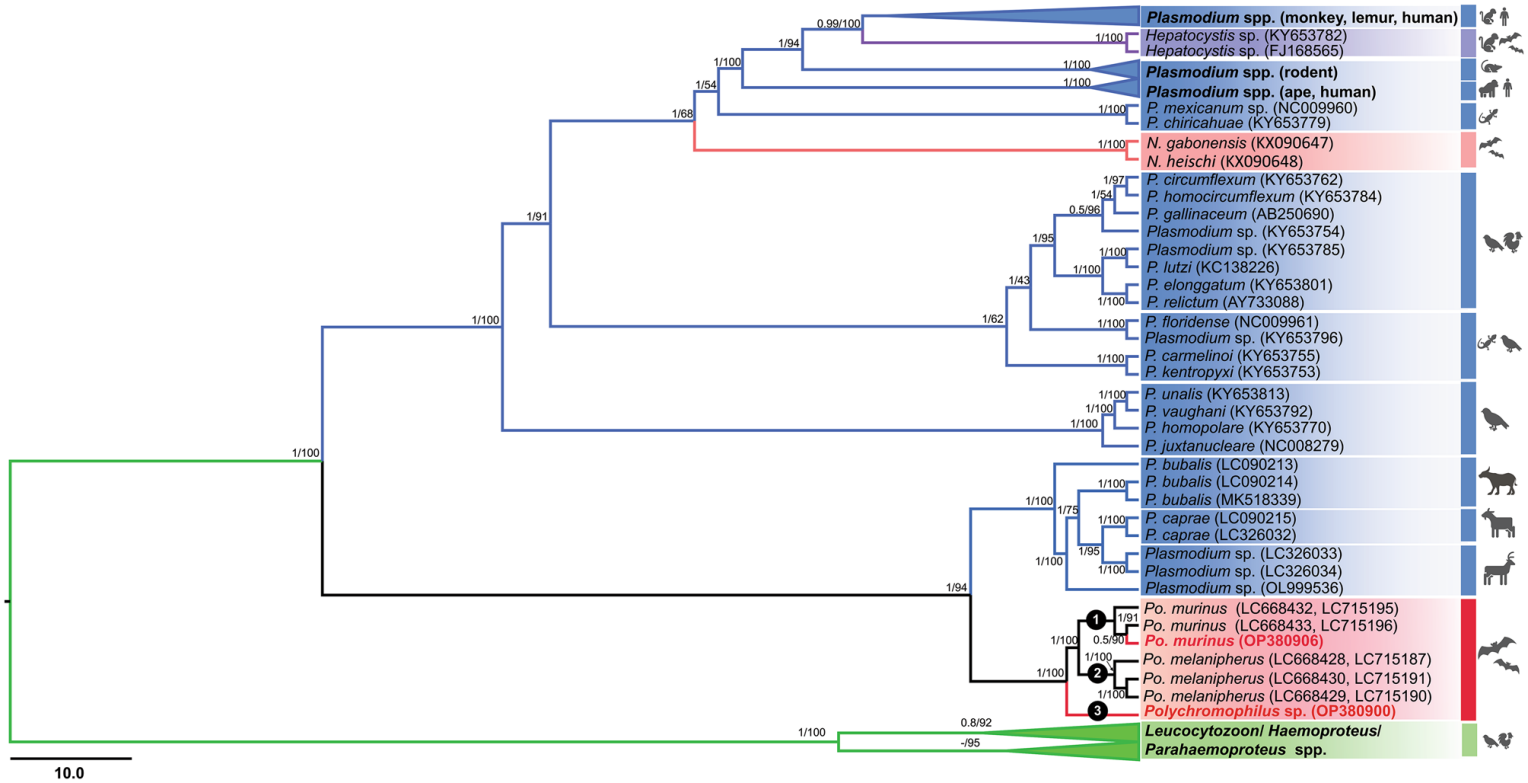
A phylogenetic tree inferred from mitochondrial genome sequences (6227 bp including gaps) of haemosporidian parasites, using a total of 95 sequences. The *Polychromophilus* sequences obtained in this study are visually distinguished by the color red. The distinct subclades within the *Polychromophilus* genus are labeled as 1, 2, and 3 for ease of reference. Bayesian posterior probabilities (BPP) and maximum likelihood bootstrap values (BV) are reported at each node, indicating the level of support for the inferred relationships.

**Figure 3:**
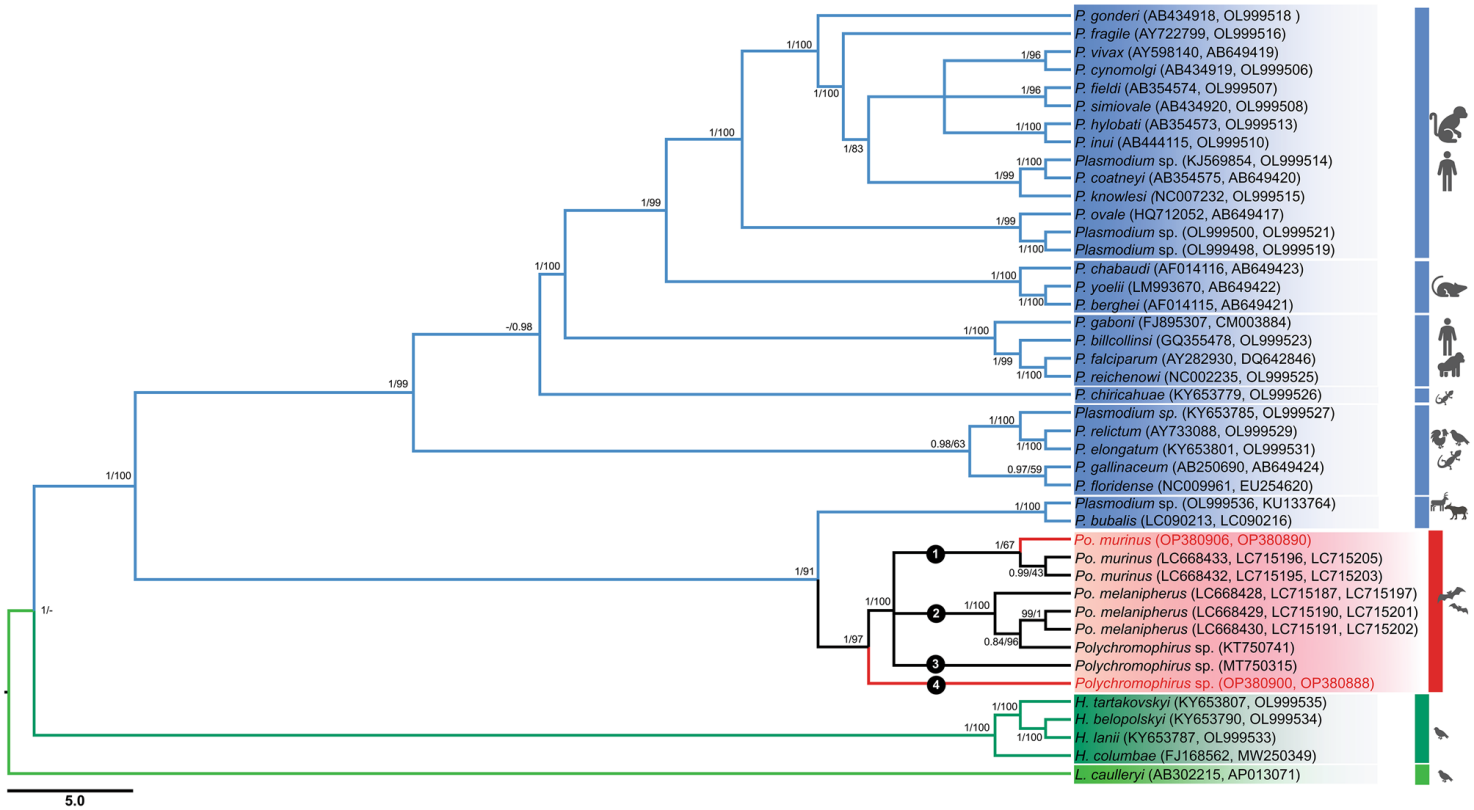
A phylogenetic tree was inferred from the mitochondrial genome sequences and *clpC* of haemosporidian parasites, using a total of 43 sequences and 6604 bp including gaps. The sequences obtained in this study are visually emphasized in red, allowing for easy identification. The distinct clades within the *Polychromophilus* genus are denoted by circled numbers ranging from 1 to 4. Bayesian posterior probabilities (BPP) and ML bootstrap values (BV) are shown at each node.

The phylogenetic analysis based on whole mitochondrial genome sequences revealed the monophyletic nature of *Polychromophilus* parasites, forming a distinct clade with ungulate *Plasmodium* parasites and displaying robust support (BPP/BV = 1/94) (Fig. 2). Within this clade, the *Polychromophilus* parasites from this study exhibited distinct positioning. *Polychromophilus murinus* from this study clustered with previously reported *Po. murinus* in clade 1, while a putatively new *Polychromophilus* species found in this study occupied clade 3, clearly separate from *Po. melanipherus* in clade 2. Furthermore, the phylogenetic analysis integrating the *clpC* gene along with whole mitochondrial genome sequences produced a similar topology, confirming the monophyly of *Polychromophilus* parasites with ungulate *Plasmodium* parasites (BPP/BV = 1/91) (Fig. 3). Notably, the *Polychromophilus* parasite identified in *H. gentilis* bats in this study formed a distinct clade that was dissimilar from both *Po. murinus* and *Po. melanipherus* (BPP/BV = 1/97), as well as from *Polychromophilus* parasites in *Sc. kuhlii* bats (BPP/BV = 1/100).

### Estimating divergence times of *Polychromophilus* and ungulate malaria parasites through whole mitochondrial genome sequences

**Figure 4:**
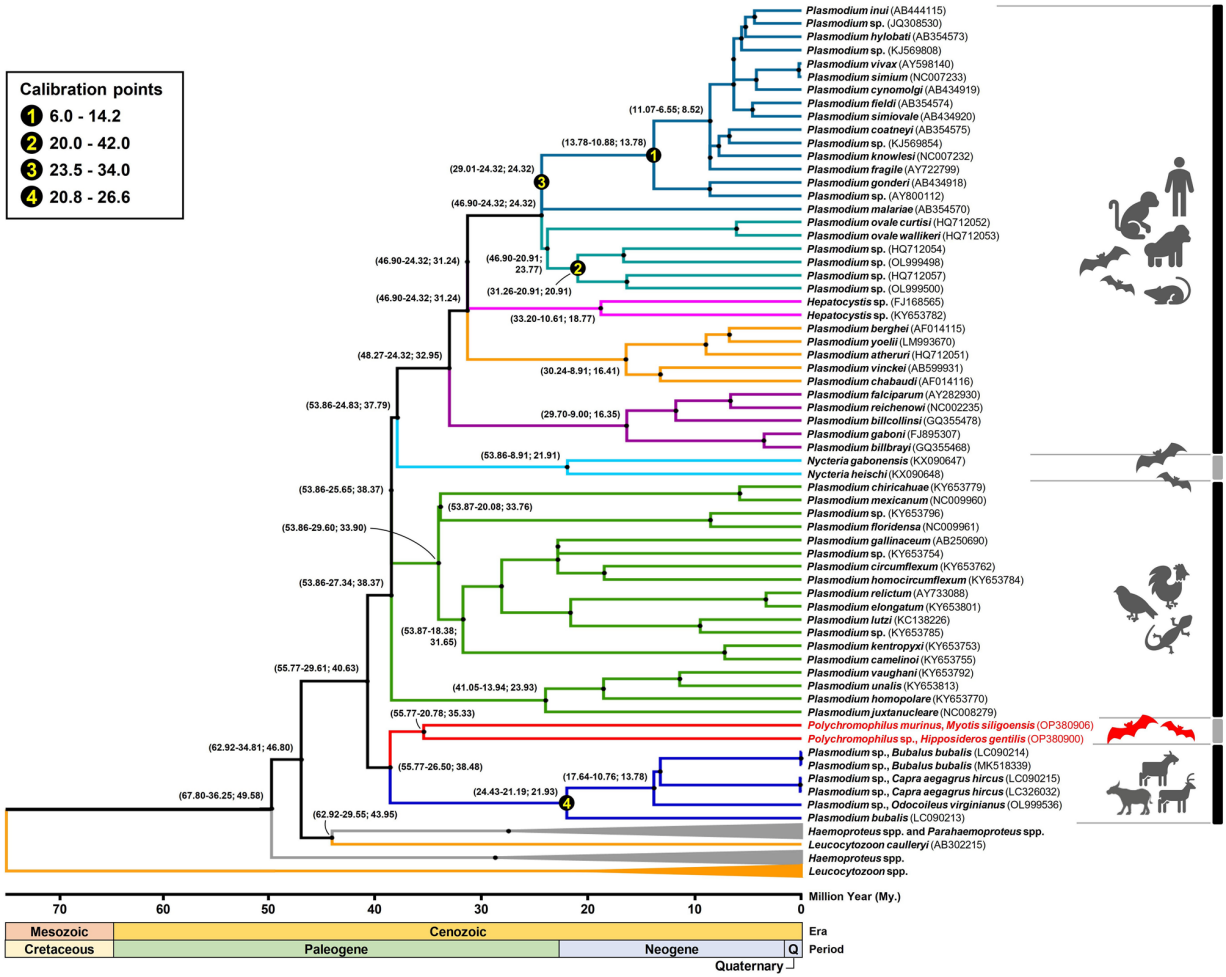
The Time Tree of divergence dating analyses using whole mitochondrial genome sequences of haemosporidian parasites. The analysis was conducted using BEAST package v2.7.5. Divergence times were estimated based on fossil evidence, including the splits of African Old-World monkeys and Asian Old-World monkeys (6–14.2 Mya) as well as the Indriidae–Lemuridae split (20–42 Mya). The divergence between human malaria *P. malariae* and monkey parasites was estimated to be between 23.5 and 34 Mya, while the origins of malaria parasites in Bovidae-Moschidae-Cervidae occurred between 20.8 and 26.6 Mya. The horizontal scale represents time in millions of years. The numbers in parentheses at each node indicate the maximum and minimum estimated divergence times in Mya, along with the node age. The *Polychromophilus* sequences obtained in this study are highlighted in red.

According to the estimated divergence dating (Fig. 4), the time to the most recent common ancestor (TMRCA) between the common ancestor of bat *Polychromophilus* and ungulate *Plasmodium* parasites and the other haemosporidian parasites in sauropsids and mammals ranged from 29.61 to 55.77 million years ago (Mya) with a 95% credibility interval (CrI), and a node age of 40.63 Mya, placing this TMRCA in the Paleogene period prior to their divergence. *Polychromophilus* shares a common ancestor with ungulate malaria parasites, dating back approximately 26.50 to 55.77 Mya (node age = 35.33 Mya). The divergence between *Po. murinus* and the potentially new *Polychromophilus* species identified in this study occurred between 20.78 and 55.77 Mya (node age = 35.33 Mya).

## Discussion

The mitochondrial genome of *Polychromophilus* exhibits a size range of 5,994 to 6,001 base pairs (bp) and contains three protein-coding genes (PCGs), resembling the characteristics observed in haemosporidian parasites of the genus *Plasmodium*. Low genetic divergence among distinct haplotypes of *Polychromophilus* from the same bat host suggests that they belong to the same parasite species, a finding similar to what is observed in *Plasmodium* lineages found in lemurs ^20^. Through BLASTN similarity searches utilizing the whole mitochondrial genome sequences of *Polychromophilus* species obtained in this study (accession no. OP380899-903), a relatively low percent identity (92.7% to 92.8%) and 99% query coverage were observed when compared to the *P. relictum* bird malaria parasite (accession no. AY733090). This finding can be attributed to the limited availability of deposited data on the complete mitochondrial genome of *Polychromophilus.* In a previous study focused on bat-infecting haemosporidian parasites^23^, relatively short *cytb* sequences (500 bp long) were utilized, resulting in inconclusive deductions and an inability to identify parasite species for several sequences. In contrast, the inclusion of additional data, encompassing whole mitochondrial genome sequences (5994–6001 bp long) and apicoplast *clpC* gene sequences (431 bp), enabled a more comprehensive examination of *Polychromophilus* species. Based on the current analysis incorporating publicly available *Polychromophilus* sequences, the *Polychromophilus*-related sequences identified in this study can be classified into four distinct species. These species include *Po. melanipherus, Po. murinus, Polychromophilus* parasites previously reported in *Scotophilus kuhlii* bats from Thailand (accession no. MT750315)^24^, and *Polychromophilus* originating from *H. gentilis* bats in this study, each supported by Bayesian posterior probabilities (BPP) and ML bootstrap values (BV) of 1/100, respectively. *Polychromophilus melanipherus* has been detected in miniopterid and emballonurid bats^23,31^, while *Polychromophilus murinus* identified in the present study appears to be confined to vespertilionid bats, aligning with previous reports^31^. The unassigned *Polychromophilus* species identified from *H. gentilis* in this study forms a distinct clade separate from the aforementioned species. Further investigations are required to determine the bat host specificity of this potentially new *Polychromophilus* species. Nonetheless, the diversity of *Polychromophilus* parasites in Thai bats seems to be more extensive than initially presumed. Regrettably, previous descriptions of *Polychromophilus* species, namely *Po. corradetti*, *Po. adami,* and *Po. deanei*, in bats have encountered controversy, lacking molecular data to facilitate comparisons with the described morphological characters^29,30^. In light of the present findings concerning the unassigned *Polychromophilus* species, the authors propose that additional information, encompassing investigations into exoerythrocytic and erythrocytic developments, a broader range of genetic markers (including nuclear genes), and identification of the vector, is essential to definitively support the classification of this potential novel species.

The phylogenetic analysis conducted in this study, based on nucleotide sequences of complete mitochondrial genomes, provides robust evidence that bat *Polychromophilus* and ungulate *Plasmodium* parasites constitute a monophyletic clade. This conclusion is strongly supported by Bayesian posterior probabilities (BPP) and maximum likelihood bootstrap values (ML-BV) of 1/94. These results align with a previous investigation by Galen et al.^10^, in which 21 nuclear protein-coding genes were employed, yielding BPP and ML-BV values of 1 and > 70, respectively. Moreover, the findings in this study are consistent with several earlier studies, which collectively suggest that the genus *Plasmodium*, housing species found in ungulates, birds, reptiles, and other mammals, does not exhibit monophyly^7,15^. The authors estimated that the divergence between the clade comprising bat *Polychromophilus* and ungulate *Plasmodium* and the clade encompassing other *Plasmodium* species occurred between 29.61 and 55.77 million years ago (Mya), with a node age of 40.63 Mya. However, the estimation in this study does not align completely with the findings of a previous study by Pacheco et al.^20^, which proposed a divergence range of 40.63 to 59.69 Mya. Pacheco et al.^20^ applied a range of 16–28.1 Mya, suggested for the origin of Bovinae-Antilopinae, as a calibration point for the time to the most recent common ancestor (TMRCA) of ungulate *Plasmodium*. It is important to note that both Bovinae and Antilopinae belong to the Bovidae family, which includes the water buffalo and the goat. On the other hand, the deer, the host of *Plasmodium odocoilei*, belongs to the Cervidae family. Therefore, this study contends that it is more appropriate to employ the TMRCA of Bovidae-Cervidae, estimated to be 20.8–26.6 Mya, for calibration purposes. Nevertheless, the values used by the authors fall within the range utilized by Pacheco et al.^20^, indicating that this discrepancy may not have a significant impact on the estimation of the divergence time between the clade containing bat *Polychromophilus* and ungulate *Plasmodium* and the clade encompassing other *Plasmodium* species. The discrepancy observed may be attributed to the inclusion of *Polychromophilus* data in the current analysis, which could have provided additional insights into the divergence pattern.

The authors estimated that the divergence between bat *Polychromophilus* and ungulate malaria parasites occurred approximately 26.50 to 55.77 million years ago (Mya), with a node age of 38.48 Mya. To gain further insights, the authors consulted the TimeTree of Life database (https://www.timetree.org), which provided an estimated pairwise divergence time of 68.4–82.6 Mya (median time 81 Mya) for the common ancestors of Chiroptera (bats) and Bovidae-Cervidae (ungulates). Based on this information, it is unlikely that bat *Polychromophilus* and ungulate *Plasmodium* species co-evolved with their respective host groups. The estimated divergence time of *Polychromophilus* aligns with the presence of bat hosts during the early Eocene period, around 50 Mya, as indicated by the discovery of the oldest known bat fossil in southwest Wyoming, a state in the United States of America^32^. On the other hand, the estimated time to the most recent common ancestor (TMRCA) of the ungulate *Plasmodium* species (between 21.19 and 24.43 Mya) is more recent compared to the estimates for the bat *Polychromophilus* clade (between 22.78 and 55.77 Mya). This observation leads to the speculation that ungulates may have become hosts of *Plasmodium* through a host-switching event from bats. However, it is important to note that the data for a mousedeer, which belongs to the Tragulidae family and serves as a host for *Plasmodium traguli,* was not included in this study. The Tragulidae family is part of the Ruminantia suborder, which includes the Bovidae and Cervidae families. The TMRCA of Tragulidae and Pecora (the infraorder that includes Bovidae and Cervidae) was estimated at 38.3–49.8 Mya (median 45 Mya) according to the TimeTree of Life. The inclusion of *P. traguli* sequences may provide additional insights into the evolution of ungulate malaria parasites. Furthermore, it would be beneficial to obtain additional fossil records representing both the oldest and youngest ages of bats in the Chiroptera order to achieve a more precise estimation of the divergence time between these hosts and their respective parasite species. Additionally, including more ungulate fossils in the analysis could contribute to a better understanding of the divergence time between these host groups and, consequently, the divergence time for their respective parasite species.

## Conclusions

This study successfully obtained 11 complete mitochondrial genome sequences of *Polychromophilus* parasites, ranging in length from 5994 to 6001 bp. These genomes consist of three protein-coding genes (PCGs), seven small subunit ribosomal RNA genes (SSU rRNA), 12 large subunit ribosomal RNA genes (LSU rRNA), and seven miscellaneous RNA genes. Phylogenetic analysis by the authors revealed *Polychromophilus* in bats and *Plasmodium* in ungulates form a single clade, indicating a close evolutionary relationship between these parasite groups. The estimated divergence time between them falls within the range of 26.50 to 55.77 million years ago (Mya), with a node age estimated at 38.48 Mya. These findings provide further evidence that the genus *Plasmodium*, encompassing species found in various host groups such as ungulates, birds, reptiles, and other mammals, does not constitute a monophyletic group. This study contributes to a better understanding of the evolutionary history and diversity of these parasites, shedding light on their complex relationships across different host taxa.

## Methods

### Blood samples, DNA extraction, mitochondrial genome amplification and sequencing

Following established procedures, bat blood samples were collected from designated sampling sites in Kanchanaburi, located in western Thailand, during the years 2019 and 2021^23,33,34^. For this study, four bat samples (2 *Myotis siligoensis* and 2 *Hipposideros gentilis*) were utilized. These samples had previously tested positive for *Polychromophilus* parasites using specific oligonucleotide primers in a previous study^23^. Photographs of blood smears derived from these samples are depicted in Fig. S1. Genomic DNA was extracted from the collected whole blood samples using a NucleoSpin Blood Kit (Macherey–Nagel, Germany) following the manufacturer's instructions. Subsequently, PCR amplification of the partial sequence of the *cytb* gene of haemosporidian parasites and sequencing procedures were carried out as previously described^23^. The circular mitochondrial genomes of *Polychromophilus* parasites were amplified through inverse nested PCR using Takara LA Taq polymerase (Takara Mirus Bio Inc., Japan), following the manufacturer's instructions. Primers were designed based on the *cytb* sequences of *Polychromophilus* obtained in a previous study by the authors^23^. The primary PCR was conducted using the primers PolycMtCytOF (CTTACATTTACAAGGTAGCACTAATCCTTTAGG) and PolycMtCytOR (GTTGGGTCACTTACAAGATATCCACC). The PCR cycling conditions included an initial denaturation at 94 °C for 1 min, followed by 40 cycles of denaturation at 94 °C for 30 s, annealing at 54 °C for 30 s, and extension at 67 °C for 7 min. This was followed by a final extension at 72 °C for 10 min. Three microliters of the primary PCR product were used as a template for a nested PCR reaction using the primers PolycMtCytIF (GCTACTCCATTACATATAGTTCCAGAATGG) and PolycMtCytIR (CACCGCATATCCATGATACAAGACC). The PCR cycling conditions for the nested PCR were identical to those of the primary PCR. At least two independent amplified products, approximately 6 kb in size, were purified using the NucleoSpin Gel and PCR Clean-up mini-kit (Macherey–Nagel, Germany) and cloned into the pGEM-T easy vector (Promega, USA). For each product, at least four clones were sequenced. The Illumina Paired-End Sequencing platform was employed, utilizing the MiSeq Reagent Kit v2 cluster chemistry and flow cells with a read length of 2 × 25 bp (Illumina, USA). The execution of Illumina sequencing procedures was carried out by U2Bio, an organization based in South Korea (https://www.u2bio.com). The amplification of the partial *clpC* gene was carried out using the specific primers clpc-F (GGTAAAACTGAATTAGCAAAAATATTA) and clpc-R (GGACGAGCTCCATATAAAGGAT), as described by Martinsen et al.^7^. The PCR cycling conditions involved an initial denaturation step at 94 °C for 4 min, followed by 35 cycles of denaturation at 94 °C for 20 s, annealing at 50 °C for 20 s, and extension at 68 °C for 1 min. A final extension step was performed at 68 °C for 7 min. Subsequently, the 674-bp PCR products were excised from the agarose gel and purified using the NucleoSpin Gel and PCR Clean-up mini-kit in preparation for Sanger DNA sequencing.

### Sequence annotation and visualization of the mitochondrial genomes

The completion of the whole mitochondrial genome sequences involved the integration of previously obtained *cytb* sequences, which were subsequently annotated and analyzed by comparison with reference sequences from various haemosporidian parasites, including *P. bubalis* (LC090213) and *P. caprae* (LC090215). The annotation process utilized tools such as NCBI ORF finder (https://www.ncbi.nlm.nih.gov/orffinder/) and MITOS WebServer. To validate the obtained sequences, the authors of this study conducted BLASTN searches against the GenBank database, which is a widely recognized and comprehensive repository of genetic information available online at https://blast.ncbi.nlm.nih.gov/Blast.cgi. Alignment of the *Polychromophilus* mitochondrial genome sequences obtained in this study was performed using MUSCLE v3.8.425, comparing them with sequences from *P. falciparum* and *P. gallinaceum* (M76611 and AB250690). The identification of genes, including the fragmented SSU rRNA and LSU rRNA, was based on the annotation of the *P. falciparum* mitochondrial genome sequence^35^. The resulting genome maps were visualized using SnapGene® Viewer 6.0.6. Additionally, the GC content and GC skew were determined using an online tool (https://proksee.ca/projects/new).

### Pairwise evolutionary divergences and phylogenetic analyses

Pairwise evolutionary divergences were estimated among the *Polychromophilus* lineages found in bats using mitochondrial genomes, applying the Nei-Gojobori method with Jukes-Cantor correction as implemented in MEGA X (https://www.megasoftware.net/). In this study, mitochondrial genome sequences of *Polychromophilus* sp., ranging from 5994 to 6001 bp in length, were obtained (as listed in Table 1) and aligned with sequences from the GenBank database (Table S4). The final dataset, consisting of 95 haemosporidian sequences and 6,227 bp, including gaps, was subjected to phylogenetic analysis. The maximum likelihood (ML) method with IQ-TREE^36^ was employed to construct phylogenetic trees, utilizing the best-fitting model determined by ultrafast bootstrap approximation (UFBoot) with 1000 replicates. Bayesian inference (BI) analysis was performed using MrBayes 3.2.7a via the CIPRES Science Gateway (https://www.phylo.org/). The General Time Reversible (GTR) + I + G model, selected based on the Akaike Information Criterion (AIC) in ModelTest 2.1.4, was utilized for the BI analysis. Furthermore, a phylogenetic tree for *Polychromophilus* was constructed using concatenated whole mitochondrial sequences and the *clpC* gene of the apicoplast (as listed in Table S5 and the data obtained in this study; OP380887-90, OP380899-909). This analysis included *Polychromophilus* sequences from bats in Thailand, Cambodia, Japan, Switzerland, Madagascar, Kenya, Gabon, and Guinea. A total of 43 sequences and 6604 bp including gaps were analyzed using ML and BI methods, as described above. The phylogenetic trees were visualized using FigTree v1.4.4. ML consensus trees were employed for all phylogenetic inferences in this study.

### Divergence time estimation of *Polychromophilus*

A total of 88 nucleotide sequences for the whole mitochondrial genome of *Polychromophilus* (2 sequences from this study: accession numbers OP380900 and OP380906) and other haemosporidian parasites (86 sequences as listed in Table S4), including *Plasmodium*, *Hepatocystis*, *Nycteria*, *Haemoproteus*, *Parahaemoproteus*, and *Leucocytozoon* genera were aligned using MUSCLE v3.7 through the CIPRES Science Gateway. All nucleotide sequences were aligned and trimmed, which left a total length of 6123 bp, including gaps, and subsequently imported to BEAUti v2.7.5^37^ as an input for the evolutionary mode and rate calculations. The best-fitting nucleotide substitution model (site model) was selected using BEAST model test^38^ available in BEAUti v2.7.5, and the evolution rate was then estimated using a fast relaxed clock log normal model. Meanwhile, a Yule model was chosen as tree prior for species-level speciations and datings, as suggested by Suchard et al.^39^.

To estimate the divergence times for the *Polychromophilus* clade, this study incorporated four calibration points into the analysis. The first two calibration points relied on the ages of vertebrate hosts' fossils obtained from publicly available sources such as the TimeTree of Life (http://www.timetree.org) and the Paleobiology Database (https://paleobiodb.org). The first calibration point suggested that the divergence between malaria parasites found in African Old-World monkeys (accession numbers AB434918 and AY800112) and those found in Asian Old-World monkeys occurred approximately 6 to 14.2 million years ago (Mya)^40,41^. Similarly, the second calibration point indicated that the origin of malaria parasites in Indriidae (accession numbers OL999500 and HQ712057) and Lemuridae (accession numbers OL999498 and HQ712054) dated back to a range of 20 to 42 Mya^20,42,43^. Furthermore, the last two calibration points were established based on molecular dating analyses. The divergence between human malaria *P. malariae* (accession number AB354570) and malaria parasites in Macaca monkeys was estimated to have occurred between 23.5 and 34 Mya, as investigated by Pacheco et al.^20^. Similarly, malaria parasites originating in ungulates (Bovidae-Moschidae-Cervidae) were found to have diverged between 20.8 and 26.6 Mya, which aligned with the origin of their hosts^44^. To estimate the divergence times based on the aforementioned calibration points and settings (see supplementary XML file), the Bayesian method implemented in the BEAST package v2.7.3^37^ was employed through the CIPRES Science Gateway. The analysis involved running 100,000,000 MCMC generations and sampling every 10,000th generation. The convergence of the Markov chain and estimated sample sizes (ESS) were assessed using Tracer v1.7.1^45^. The first 10% of MCMC generations were discarded as burn-in, and the maximum clade credibility tree was constructed using TreeAnnotator v2.7.5. Finally, the time tree depicting the estimated divergence dating of the *Polychromophilus* and other haemosporidian parasite clades was visualized using FigTree v1.4.4.

### Ethical statement and permits

The samples in this study were collected according to the protocol approved by the Chulalongkorn University Faculty of Veterinary Science Biosafety Committee (IBC No. 2031032) and the Institutional Animal Care and Use Committee for the university regulations and policies governing the care and use of laboratory animals (IACUC No. 1931090). Bats were captured under permits issued by Thailand’s Department of National Parks, Wildlife, and Plant Conservation (DNP 0909.6/22928 and DNP 0907.4/29038). This study was reported in accordance with ARRIVE guidelines (https://arriveguidelines.org).

### Supplementary Information

Supplementary Figure S1.

Supplementary Information.

Supplementary Table S1.

Supplementary Table S2.

Supplementary Table S3.

Supplementary Table S4.

Supplementary Table S5.

## Data Availability

The nucleotide sequences of the *clpC* gene partial sequences and the whole mitochondrial genome sequences obtained in this study were deposited in the GenBank database (https://www.ncbi.nlm.nih.gov/nuccore) under accession numbers OP380887-88 (*clpC*, *Po.* sp*.*); OP380889-90 (*clpC*, *Po. murinus*); OP380899-903 (mitogenome, *Po.* sp.); OP380904-909 (mitogenome, *Po. murinus*).
