# Supplementary material for: Complete mitochondrial genome analyses confirm that bat Polychromophilus and ungulate Plasmodium constitute a distinct clade independent of other Plasmodium species
Source: Sci Rep. 2023 Nov 20;13:20258. doi: 10.1038/s41598-023-45551-z (PMC10662395; doi:10.1038/s41598-023-45551-z)
Supplement: Supplementary file 1 — Supplementary Figure S1. [file 41598_2023_45551_MOESM1_ESM.docx]

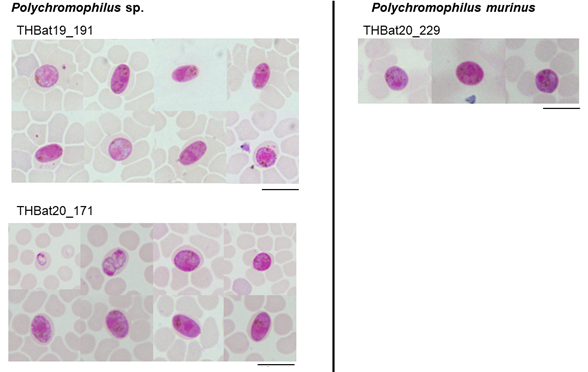


**Figure S1.** Photographs of blood smears featuring the parasites employed in this study. These images depict different forms of the gametocyte stage of *Polychromophilus*. The provided scale bar corresponds to a length of 10 μm. The specific parasite morphology associated with the sample ID ThBat20_242 bat (*Po. murinus*) was not found within the blood smear examined.
