# Supplementary material for: Complete mitochondrial genome analyses confirm that bat Polychromophilus and ungulate Plasmodium constitute a distinct clade independent of other Plasmodium species
Source: Sci Rep. 2023 Nov 20;13:20258. doi: 10.1038/s41598-023-45551-z (PMC10662395; doi:10.1038/s41598-023-45551-z)
Supplement: Supplementary file 3 — Supplementary Table S1. [file 41598_2023_45551_MOESM3_ESM.docx]

**Table S1** Pairwise distances among nucleotide sequences obtained from individual bats.

|  | Pairwise distances (d ± standard error) | | | |
| --- | --- | --- | --- | --- |
| Bat ID & Clone No. | C1 | C2 | C3 | C4 |
| THBat20_171_C1 |  | 0.002 | 0.001 | 0.002 |
| THBat20_171_C2 | 0.005 |  | 0.002 | 0.002 |
| THBat20_171_C3 | 0.002 | 0.004 |  | 0.001 |
| THBat20_171_C4 | 0.002 | 0.004 | 0.001 |  |

|  | Pairwise distances (d ± standard error) | |
| --- | --- | --- |
| Bat ID & Clone No. | C1 | C2 |
| THBat20_229_C1 |  | 0.002 |
| THBat20_229_C2 | 0.006 |  |

|  | Pairwise distances (d ± standard error) | | | |
| --- | --- | --- | --- | --- |
| Bat ID & Clone No. | C1 | C2 | C3 | C4 |
| THBat20_242_C1 |  | 0.002 | 0.003 | 0.004 |
| THBat20_242_C2 | 0.005 |  | 0.002 | 0.003 |
| THBat20_242_C3 | 0.007 | 0.003 |  | 0.003 |
| THBat20_242_C4 | 0.010 | 0.007 | 0.009 |  |

Pairwise evolutionary divergences were estimated among the *Polychromophilus* lineages found in bats using mitochondrial genomes, applying the Nei-Gojobori method with Jukes-Cantor correction as implemented in MEGA X (https://www.megasoftware.net/).
