# Supplementary material for: Complete mitochondrial genome analyses confirm that bat Polychromophilus and ungulate Plasmodium constitute a distinct clade independent of other Plasmodium species
Source: Sci Rep. 2023 Nov 20;13:20258. doi: 10.1038/s41598-023-45551-z (PMC10662395; doi:10.1038/s41598-023-45551-z)
Supplement: Supplementary file 4 — Supplementary Table S2. [file 41598_2023_45551_MOESM4_ESM.docx]

**Table S2.** Annotation of the *Polychromophilus* mitochondrial genome using *P. falciparum* (M76611) as a reference

| **Name** | **Product** | ***P. falciparum* (M76611)** | **No. Sites (M76611)** | **From - to:** | **No. Sites in alignment** | **No. Sites (*Polychromophilus*)** |
| --- | --- | --- | --- | --- | --- | --- |
| intergenic-region |  | 1-99 | 99 | 1-99 | 99 | 99 |
| rns | Small Subunit Ribosomal RNA: fragment number 4 (synonym=RNA9) | 100-165 | 67 | 100-166 | 67 | 66-67 |
| intergenic-region |  | 166-205 | 40 | 167-210 | 44 | 42-44 |
| rnl | Large Subunit Ribosomal RNA: fragment number 4 (synonym=LSUC) | 206-221 | 16 | 211-226 | 16 | 16 |
| intergenic-region |  | 222-282 | 61 | 227-288 | 62 | 61 |
| rnl | Large Subunit Ribosomal RNA: fragment number 11 (synonym=LSUG) | 283-389 | 107 | 289-395 | 107 | 107 |
| rns | Small Subunit Ribosomal RNA: fragment number 3 (synonym=SSUB) | 390-502 | 113 | 396-508 | 113 | 113 |
| intergenic-region |  | 503-505 | 3 | 509-512 | 4 | 3-4 |
| rnl | Large Subunit Ribosomal RNA: fragment number 5 (synonym=RNA1) | 506-606 | 101 | 513-613 | 101 | 101 |
| intergenic-region |  | 607-624 | 18 | 614-636 | 23 | 23 |
| rnl | Large Subunit Ribosomal RNA: fragment number 12 (synonym=RNA10) | 625-724 | 100 | 637-736 | 100 | 97-98 |
| ***cox3*** | ***cytochrome c oxidase subunit 3*** | **725-1487** | **763** | **737-1498** | **762** | **761** |
| misc_feature | signal mot; copy number 3 | 1474-1488 | 15 | 1486-1505 | 20 | 19-20 |
| intergenic-region |  | 1489-1500 | 13 | 1506-1517 | 12 | 12 |
| rnl | Large Subunit Ribosomal RNA: fragment number 10 (synonym=LSUF) | 1501-1630 | 130 | 1518-1648 | 131 | 130-131 |
| intergenic-region |  | 1631-1649 | 19 | 1649-1671 | 23 | 18-23 |
| rns | Small Subunit Ribosomal RNA: fragment number 6 (synonym=SSUE) | 1650-1688 | 39 | 1672-1711 | 40 | 40 |
| intergenic-region |  | 1689-1696 | 8 | 1712-1719 | 8 | 8 |
| rnl | Large Subunit Ribosomal RNA: fragment number 2 (synonym=RNA2) | 1697-1763 | 67 | 1720-1787 | 68 | 67 |
| intergenic-region |  | 1764-1830 | 67 | 1788-1857 | 70 | 69-70 |
| rnl | Large Subunit Ribosomal RNA: fragment number 6 (synonym=RNA3) | 1831-1910 | 80 | 1858-1937 | 80 | 80 |
| intergenic-region |  | 1911-1915 | 5 | 1938-1942 | 5 | 4 |
| rns | Small Subunit Ribosomal RNA: fragment number 1 (synonym=SSUA) | 1916-2023 | 108 | 1943-2050 | 108 | 108 |
| intergenic-region |  | 2024-2035 | 12 | 2051-2064 | 14 | 14 |
| misc_feature | signal mot; copy number 1 | 2036-2050 | 15 | 2065-2081 | 17 | 17 |
| ***cox1*** | ***cytochrome c oxidase subunit I*** | **2037-3479** | **1443** | **2069-3502** | **1434** | **1434** |
| misc_feature | signal mot; copy number 2 | 3478-3493 | 16 | 3503-3521 | 19 | 12-19 |
| ***cytb*** | ***cytochrome b (apocytochrome b)*** | **3480-4624** | **1146** | **3522-4664** | **1143** | **1143** |
| rnl | Large Subunit Ribosomal RNA: fragment number 3 (synonym=LSUB) | 4594-4618 | 25 | 4636-4660 | 25 | 25 |
| misc_RNA | RNA4 | 4625-4696 | 72 | 4667-4738 | 72 | 71-72 |
| intergenic-region |  | 4697-4716 | 20 | 4739-4758 | 20 | 20 |
| misc_RNA | RNA5 | 4717-4802 | 86 | 4759-4845 | 87 | 87 |
| misc_RNA | RNA6 | 4803-4865 | 63 | 4846-4913 | 68 | 58-68 |
| intergenic-region |  | 4866-4886 | 21 | 4914-4934 | 21 | 21 |
| misc_RNA | RNA12 | 4887-4945 | 59 | 4935-4993 | 59 | 59 |
| intergenic-region |  | 4946-4995 | 50 | 4994-5050 | 57 | 54-57 |
| rnl | Large Subunit Ribosomal RNA: fragment number 9 (synonym=RNA13) | 4996-5025 | 30 | 5051-5080 | 30 | 30 |
| rnl | Large Subunit Ribosomal RNA: fragment number 1 (synonym=LSUA) | 5026-5201 | 176 | 5081-5256 | 176 | 176 |
| misc_RNA | RNA7 | 5202-5283 | 82 | 5257-5339 | 83 | 83 |
| misc_RNA | RNA11 | 5284-5378 | 95 | 5340-5436 | 97 | 96-97 |
| rns | Small Subunit Ribosomal RNA: fragment number 5 (synonym=SSUD) | 5379-5446 | 68 | 5437-5504 | 68 | 68 |
| rns | Small Subunit Ribosomal RNA: fragment number 7 (synonym=SSUF) | 5447-5507 | 61 | 5505-5565 | 61 | 60 |
| misc_RNA | RNA14 | 5508-5562 | 55 | 5566-5620 | 55 | 55 |
| intergenic-region |  | 5563-5576 | 14 | 5621-5634 | 14 | 14 |
| rnl | Large Subunit Ribosomal RNA: fragment number 8 (synonym=LSUE) | 5577-5771 | 195 | 5635-5830 | 196 | 196 |
| rnl | Large Subunit Ribosomal RNA: fragment number 7 (synonym=LSUD) | 5772-5854 | 83 | 5831-5913 | 83 | 83 |
| rns | Small Subunit Ribosomal RNA: fragment number 2 (synonym=RNA8) | 5855-5955 | 101 | 5914-6014 | 101 | 100-101 |
| intergenic-region |  | 5956-5967 | 12 | 6015-6026 | 12 | 10-11 |
