# Supplementary material for: Complete mitochondrial genome analyses confirm that bat Polychromophilus and ungulate Plasmodium constitute a distinct clade independent of other Plasmodium species
Source: Sci Rep. 2023 Nov 20;13:20258. doi: 10.1038/s41598-023-45551-z (PMC10662395; doi:10.1038/s41598-023-45551-z)
Supplement: Supplementary file 5 — Supplementary Table S3. [file 41598_2023_45551_MOESM5_ESM.docx]

**Table S3.** Annotation of the *Polychromophilus* mitochondrial genome using *P. gallinaceum* (AB250690) as a reference

| **Name** | **Product** | ***P. gallinaceum* (AB250690)** | **No. Sites (AB250690)** | **From - to:** | **No. Sites in alignment** | **No. Sites (*Polychromophilus*)** |
| --- | --- | --- | --- | --- | --- | --- |
| rRNA | Similar to prokaryote large subunit ribosomal RNA (LS1) | 330-508 | 179 | 5046-5053, 5224-5231 | 179 | 179 |
| rRNA | Similar to prokaryote large subunit ribosomal RNA (LS4) | 700-754 | 55 | 5419-5426, 5474-5480 | 55 | 55 |
| rRNA | Similar to prokaryote small subunit ribosomal RNA (SS6) | 758-836 | 79 | 5477- 5485, 5555-5562 | 79 | 79 |
| rRNA | Similar to prokaryote large subunit ribosomal RNA (LS7) | 893-1081 | 189 | 5612-5619, 5800-5807 | 189 | 189 |
| rRNA | Similar to prokaryote large subunit ribosomal RNA (LS6) | 1085-1165 | 79 | 5804-5811, 5882-5889 | 79 | 79 |
| rRNA | Similar to prokaryote small subunit ribosomal RNA (SS3) | 1305-1377 | 73 | 28, 100 | 73 | 73 |
| rRNA | Similar to prokaryote large subunit ribosomal RNA (LS3) | 1401-1503 | 103 | 122-124, 226-277 | 103 | 103 |
| rRNA | Similar to prokaryote large subunit ribosomal RNA (LS9) | 1567-1663 | 97 | 290-291, 386-387 | 97 | 97 |
| rRNA | Similar to prokaryote small subunit ribosomal RNA (SS2) | 1667-1785 | 119 | 390-391, 508-509 | 119 | 119 |
| rRNA | Similar to prokaryote large subunit ribosomal RNA (LS4) | 1831-1865 | 35 | 555-619 | 35 | 35 |
| rRNA | Similar to prokaryote large subunit ribosomal RNA (LS5) | 1872-1895 | 24 | 596- 619 | 24 | 24 |
| *CoxIII* | cytochrome c oxidase subunit III | 2019-2768 | 750 | 739-741, 1488-1490 | 750 | 750 |
| rRNA | Similar to prokaryote large subunit ribosomal RNA (LS8) | 2805-2914 | 110 | 1524-1526, 1633-1636 | 110 | 110 |
| rRNA | Similar to prokaryote small subunit ribosomal RNA (SS5) | 2944-2971 | 28 | 1661-1664, 1688-1691 | 28 | 28 |
| rRNA | Similar to prokaryote small subunit ribosomal RNA (SS1) | 3217-3326 | 110 | 1933-1937, 2024-2046 | 110 | 110 |
| *CoxI* | cytochrome c oxidase subunit I | 3339-4772 | 1432 | 2055-2059, 3488-3492 | 1432 | 1432 |
| *Cytb* | cytochrome b | 4795-5925 | 1131 | 3513-3524, 4643-4654 | 1131 | 1131 |
| rRNA | Similar to prokaryote large subunit ribosomal RNA (LS2) | 5895-5987 | 93 | 4613-4624, 4704-4716 | 93 | 93 |
