# Supplementary material for: Complete mitochondrial genome analyses confirm that bat Polychromophilus and ungulate Plasmodium constitute a distinct clade independent of other Plasmodium species
Source: Sci Rep. 2023 Nov 20;13:20258. doi: 10.1038/s41598-023-45551-z (PMC10662395; doi:10.1038/s41598-023-45551-z)
Supplement: Supplementary file 6 — Supplementary Table S4. [file 41598_2023_45551_MOESM6_ESM.docx]

| **Table S4.** Nucleotide sequences of mitochondrial genomes for phylogenetic analysis and divergence time estimation used in this study | | | | | | | |
| --- | --- | --- | --- | --- | --- | --- | --- |
| **GenBank accession no.** | | | | **Haemosporidian Parasites** | **Vertebrate host** | | **Length** |
| **mtDNA** | ***cytb*** | ***coxI*** | ***coxIII*** |  |  |  | **(bp)** |
| AB250690 |  |  |  | *Plasmodium* *gallinaceum* | *Gallus gallus domesticus* | | 6003 |
| AB299369 |  |  |  | *Leucocytozoon sabrazesi* | *Gallus gallus domesticus* | | 5935 |
| AB302215 |  |  |  | *Leucocytozoon caulleryi* | *Gallus gallus domesticus* | | 5959 |
| AB354570 |  |  |  | *Plasmodium malariae* | *Homo sapiens* | | 5968 |
| AB354573 |  |  |  | *Plasmodium hylobati* | *Hylobati moloch* | | 5973 |
| AB354574 |  |  |  | *Plasmodium fieldi* | *Macaca nemestrina* | | 5983 |
| AB354575 |  |  |  | *Plasmodium coatneyi* | *Macaca fascicularis* | | 5976 |
| AB434918 |  |  |  | *Plasmodium gonderi* | Asian Old-World monkeys | | 5989 |
| AB434919 |  |  |  | *Plasmodium cynomolgi* | Asian Old-World monkeys | | 5983 |
| AB434920 |  |  |  | *Plasmodium simiovale* | *Macaca sinica* | | 5987 |
| AB444115 |  |  |  | *Plasmodium inui* | *Macaca fascicularis* | | 5971 |
| AB599931 |  |  |  | *Plasmodium vinckei vinckei* | *Thamnomy*s sp./Grammomys sp. | | 5948 |
| AF014115 |  |  |  | *Plasmodium berghei* | *Grammomys* sp. | | 5961 |
| AF014116 |  |  |  | *Plasmodium chabaudi* | *Thamnomy*s sp. | | 5948 |
| AY282930 |  |  |  | *Plasmodium falciparum* | *Homo sapiens* | | 5949 |
| AY598140 |  |  |  | *Plasmodium vivax* | *Homo sapiens* | | 5990 |
| AY722799 |  |  |  | *Plasmodium fragile* | *M. radiata, M. mulatta, Prebyti*s spp. | | 5977 |
| AY733088 |  |  |  | *Plasmodium* *relictum* | *Spheniscus demersus* | | 5996 |
| AY800112 |  |  |  | *Plasmodium* sp. | *Mandrillus sphinx* | | 5896 |
| FJ168561 |  |  |  | *Parahaemoproteus vireonis* | *Vireo gilvus* | | 5893 |
| FJ168562 |  |  |  | *Haemoproteus columbae* | *Columba livia* | | 5988 |
| FJ168563 |  |  |  | *Leucocytozoon majoris* | *Zonotrichia leucophrys oriantha* | | 6684 |
| FJ168564 |  |  |  | *Leucocytozoon fringillinarum* | *Pipilo chlorurus* | | 5992 |
| FJ168565 |  |  |  | *Hepatocystis* sp. | *Pteropus hypomelanus* | | 6259 |
| FJ895307 |  |  |  | *Plasmodium gaboni* | Chimpanzee | | 5931 |
| GQ355468 |  |  |  | *Plasmodium billbrayi* | *Pan troglodytes* | | 5945 |
| GQ355478 |  |  |  | *Plasmodium billcollinsi* | Chimpanzee | | 5864 |
| HQ712051 |  |  |  | *Plasmodium atheruri* | *Atherurus africanus* | | 5851 |
|  |  |  |  |  |  | |  |
| **Table S4.** Nucleotide sequences of mitochondrial genomes for phylogenetic analysis and divergence time estimation used in this study (cont.) | | | | | | | |
| **GenBank accession no.** | | | | **Haemosporidian Parasites** | **Vertebrate host** | | **Length** |
| **mtDNA** | ***cytb*** | ***coxI*** | ***coxIII*** |  |  | | **(bp)** |
| HQ712052 |  |  |  | *Plasmodium ovale-curtisi* | *Homo sapiens* | | 5851 |
| HQ712053 |  |  |  | *Plasmodium ovale-wallikeri* | *Homo sapiens* | | 5855 |
| HQ712054 |  |  |  | *Plasmodium* sp. | *Hapalemur griseus griseus* | | 5849 |
| HQ712057 |  |  |  | *Plasmodium* sp. | *Indri indri* | | 5564 |
| JQ308530 |  |  |  | *Plasmodium* sp. | *Pongo pygmaeus* | | 5868 |
| KC138226 |  |  |  | *Plasmodium* *lutzi* | *Turdus fuscater*/Great thrush | | 5889 |
| KF479480 |  |  |  | *Leucocytozoon quynzae* | *Heliangelus amethysticollis* | | 5868 |
| KJ569808 |  |  |  | *Plasmodium* sp. | *Pongo pygmaeus morio* | | 5862 |
| KJ569854 |  |  |  | *Plasmodium* sp. | *Macaca nemestrina* | | 5877 |
| KM610045 |  |  |  | *Leucocytozoon* sp. | *Grallaria quitensis* | | 5867 |
| KM610046 |  |  |  | *Leucocytozoon* sp. | *Grallaria ruficapilla* | | 5896 |
| KX090647 |  |  |  | *Nycteria gabonensis* | *Rhinolophus alcyone* | | 5999 |
| KX090648 |  |  |  | *Nycteria heischi* | *Megaderma spasma* | | 5989 |
| KY653752 |  |  |  | *Haemoproteus coatneyi* | *Tangara nigroviridis* | | 5881 |
| KY653753 |  |  |  | *Plasmodium kentropyxi* | *Cnemidophorus gramivagus* | | 5895 |
| KY653754 |  |  |  | *Plasmodium* sp. | *Bubo scandiacus* | | 5893 |
| KY653755 |  |  |  | *Plasmodium carmelinoi* | *Ameiva ameiva* | | 5896 |
| KY653756 |  |  |  | *Haemoproteus multipigmentatus* | *Zenaida galapagoensis* | | 5877 |
| KY653757 |  |  |  | *Haemoproteus noctuae* | *Asio otus*/Long-eared owl | | 5878 |
| KY653758 |  |  |  | *Haemoproteus jenniae* | *Creagrus furcatus* | | 5888 |
| KY653762 |  |  |  | *Plasmodium* *circumflexum* | *Troglodytes troglodytes* | | 5900 |
| KY653763 |  |  |  | *Haemoproteus minutus* | *Turdus merula* | | 5507 |
| KY653768 |  |  |  | *Haemoproteus motacillae* | *Motacilla flava* | | 5507 |
| KY653770 |  |  |  | *Plasmodium* *homopolare* | *Zonotrichia capensis* | | 5901 |
| KY653777 |  |  |  | *Haemoproteus iwa* | *Fregata minor* | | 5889 |
| KY653779 |  |  |  | *Plasmodium chiricahuae* | *Sceloporus jarrovii* | | 5833 |
| KY653781 |  |  |  | *Leucocytozoon danilewskyi* | *Asio otus* | | 5857 |
| KY653782 |  |  |  | *Hepatocystis* sp. | *Macaca fascicularis* | | 6450 |
| **Table S4.** Nucleotide sequences of mitochondrial genomes for phylogenetic analysis and divergence time estimation used in this study (cont.) | | | | | | | |
| **GenBank accession no.** | | | | **Haemosporidian Parasites** | **Vertebrate host** | | **Length** |
| **mtDNA** | ***cytb*** | ***coxI*** | ***coxIII*** |  |  | | **(bp)** |
| KY653784 |  |  |  | *Plasmodium* *homocircumflexum* | *Lanius collurio* | | 5894 |
| KY653785 |  |  |  | *Plasmodium* sp. | *Quiscalus mexicanus* | | 5896 |
| KY653787 |  |  |  | *Haemoproteus lanii* | *Lanius collurio* | | 5887 |
| KY653790 |  |  |  | *Haemoproteus belopolskyi* | *Hippolais icterina* | | 5880 |
| KY653792 |  |  |  | *Plasmodium* *vaughani* | *Turdus merula* | | 5809 |
| KY653793 |  |  |  | *Haemoproteus pastoris* | *Sturnus vulgaris* | | 5879 |
| KY653794 |  |  |  | *Haemoproteus witii* | *Eriocnemis derbyi* | | 5668 |
| KY653795 |  |  |  | *Leucocytozoon dubreuili* | *Turdus merula* | | 5848 |
| KY653796 |  |  |  | *Plasmodium* sp. | *Plica cf. plica* | | 5893 |
| KY653797 |  |  |  | *Haemoproteus erythrogravidus* | *Zonotrichia capensis* | | 5490 |
| KY653799 |  |  |  | *Haemoproteus balmorali* | *Luscinia luscinia* | | 5881 |
| KY653801 |  |  |  | *Plasmodium* *elongatum* | *Acrocephalus scirpaceus* | | 5886 |
| KY653807 |  |  |  | *Haemoproteus tartakovskyi* | *Loxia curvirostra* | | 5885 |
| KY653811 |  |  |  | *Haemoproteus sacharovi* | *Zenaida macroura* | | 5882 |
| KY653813 |  |  |  | *Plasmodium* *unalis* | *Turdus fuscater* | | 5891 |
| LC090213 |  |  |  | *Plasmodium bubalis* | *Bubalus bubalis* | | 6002 |
| LC090214 |  |  |  | *Plasmodium* sp. | *Bubalus bubalis* | | 5987 |
| LC090215 |  |  |  | *Plasmodium* sp. | *Capra aegagrus hircus* | | 5987 |
| LC326032 |  |  |  | *Plasmodium* sp. | *Capra aegagrus hircus* | | 5987 |
| LM993670 |  |  |  | *Plasmodium yoelii* | *Thamnomys sp.* | | 6083 |
| MK518339 |  |  |  | *Plasmodium* sp. | *Bubalus bubalis* | | 5987 |
| NC_002235 |  |  |  | *Plasmodium reichenowi* | *Pan troglodytes* | | 5966 |
| NC_007232 |  |  |  | *Plasmodium knowlesi* | *M. nemestrina* | | 5957 |
| NC_007233 |  |  |  | *Plasmodium simium* | *Aotus nancymai* | | 5990 |
| NC_008279 |  |  |  | *Plasmodium juxtanucleare* | *Gallus gallus domesticus* | | 6014 |
| NC_009960 |  |  |  | *Plasmodium mexicanum* | *Sceloporus occidentalis* | | 5991 |
| NC_009961 |  |  |  | *Plasmodium floridense* | *Anolis sagrei* | | 6002 |
| OL999498 |  |  |  | *Plasmodium* sp. | *Prolemur simus* | | 5858 |
|  | | | | | | | |
| **Table S4.** Nucleotide sequences of mitochondrial genomes for phylogenetic analysis and divergence time estimation used in this study (cont.) | | | | | | | |
| **GenBank accession no.** | | | | **Haemosporidian Parasites** | **Vertebrate host** | | **Length** |
| **mtDNA** | ***cytb*** | ***coxI*** | ***coxIII*** |  |  | | **(bp)** |
| OL999500 |  |  |  | *Plasmodium* sp*.* | *Propithecus diadema* | | 5842 |
| OL999536 |  |  |  | *Plasmodium* sp. | *Odocoileus virginianus* | | 5820 |
| LC326033^*, **^ | | | | *Plasmodium* sp*.* | *Ozotocerus bezoarticus* | | 3484 |
| LC326034^*, **^ | | | | *Plasmodium* sp*.* | *Ozotocerus bezoarticus* | | 3484 |
|  | LC668428^*, **^ | LC715187^*, **^ |  | *Polychromophilus melanipherus* | *Miniopterus fuliginosus* | | 1897 |
|  | LC668429^*, **^ | LC715190^*, **^ |  | *Polychromophilus melanipherus* | *Miniopterus fuliginosus* | | 2077 |
|  | LC668430^*, **^ | LC715191^*, **^ |  | *Polychromophilus melanipherus* | *Miniopterus fuliginosus* | | 1896 |
|  | LC668432^*, **^ | LC715195^*, **^ |  | *Polychromophilus murinus* | *Myotis macrodactylus* | | 1722 |
|  | LC668433^*, **^ | LC715196^*, **^ |  | *Polychromophilus murinus* | *Myotis macrodactylus* | | 1967 |

The sequences which indicated by asterisk (*) were not included for divergence time estimation, and double asterisk (**) indicated partial sequence of mitochondrial genes.
