# Supplementary material for: Complete mitochondrial genome analyses confirm that bat Polychromophilus and ungulate Plasmodium constitute a distinct clade independent of other Plasmodium species
Source: Sci Rep. 2023 Nov 20;13:20258. doi: 10.1038/s41598-023-45551-z (PMC10662395; doi:10.1038/s41598-023-45551-z)
Supplement: Supplementary file 7 — Supplementary Table S5. [file 41598_2023_45551_MOESM7_ESM.docx]

| **Table S5.** Nucleotide sequences of mitochondrial genomes and *clpC* gene for phylogenetic analysis used in this study | | | | | | |
| --- | --- | --- | --- | --- | --- | --- |
| **GenBank accession no.** | | | | | **Haemosporidian Parasites** | **Vertebrate host** |
| **mtDNA** | ***cytb*** | ***coxI*** | ***coxIII*** | ***clpc*** |  |  |
| AB250690 |  |  |  | AB649424 | *Plasmodium* *gallinaceum* | *Gallus gallus domesticus* |
| AB302215 |  |  |  | AP013071 | *Leucocytozoon caulleryi* | *Gallus gallus domesticus* |
| AB354573 |  |  |  | OL999513 | *Plasmodium hylobati* | *Hylobati moloch* |
| AB354574 |  |  |  | OL999507 | *Plasmodium fieldi* | *Macaca nemestrina* |
| AB354575 |  |  |  | AB649420 | *Plasmodium coatneyi* | *Macaca fascicularis* |
| AB434918 |  |  |  | OL999518 | *Plasmodium gonderi* | Asian Old-World monkeys |
| AB434919 |  |  |  | OL999506 | *Plasmodium cynomolgi* | Asian Old-World monkeys |
| AB434920 |  |  |  | OL999508 | *Plasmodium simiovale* | *Macaca sinica* |
| AB444115 |  |  |  | OL999510 | *Plasmodium inui* | *Macaca fascicularis* |
| AF014115 |  |  |  | AB649421 | *Plasmodium berghei* | *Grammomys* sp. |
| AF014116 |  |  |  | AB649423 | *Plasmodium chabaudi* | *Thamnomy*s sp. |
| AY282930 |  |  |  | DQ642846 | *Plasmodium falciparum* | *Homo sapiens* |
| AY598140 |  |  |  | AB649419 | *Plasmodium vivax* | *Homo sapiens* |
| AY722799 |  |  |  | OL999516 | *Plasmodium fragile* | *M. radiata, M. mulatta* |
| AY733088 |  |  |  | OL999529 | *Plasmodium* *relictum* | *Spheniscus demersus* |
| FJ168562 |  |  |  | MW250349 | *Haemoproteus columbae* | *Columba livia* |
| FJ895307 |  |  |  | CM003884 | *Plasmodium gaboni* | Chimpanzee |
| GQ355478 |  |  |  | OL999523 | *Plasmodium billcollinsi* | Chimpanzee |
| HQ712052 |  |  |  | AB649417 | *Plasmodium ovale-curtisi* | *Homo sapiens* |
| KJ569854 |  |  |  | OL999514 | *Plasmodium* sp. | *Macaca nemestrina* |
| KY653779 |  |  |  | OL999526 | *Plasmodium chiricahuae* | *Sceloporus jarrovii* |
| KY653785 |  |  |  | OL999527 | *Plasmodium* sp. | *Quiscalus mexicanus* |
| KY653787 |  |  |  | OL999533 | *Haemoproteus lanii* | *Lanius collurio* |
| KY653790 |  |  |  | OL999534 | *Haemoproteus belopolskyi* | *Hippolais icterina* |
| KY653801 |  |  |  | OL999531 | *Plasmodium* *elongatum* | *Acrocephalus scirpaceus* |
| KY653807 |  |  |  | OL999535 | *Haemoproteus tartakovskyi* | *Loxia curvirostra* |
| LC090213 |  |  |  | LC090216 | *Plasmodium bubalis* | *Bubalus bubalis* |
| LM993670 |  |  |  | AB649422 | *Plasmodium yoelii* | *Thamnomys* sp. |
| NC002235 |  |  |  | OL999525 | *Plasmodium reichenowi* | *Pan troglodytes* |
| NC007232 |  |  |  | OL999515 | *Plasmodium knowlesi* | *M. nemestrina* |
| NC009961 |  |  |  | EU254620 | *Plasmodium floridense* | *Anolis sagrei* |
| OL999498 |  |  |  | OL999519 | *Plasmodium* sp. | *Prolemur simus* |
| OL999500 |  |  |  | OL999521 | *Plasmodium* sp*.* | *Propithecus diadema* |
|  |  |  |  |  |  |  |
| **Table S5.** Nucleotide sequences of mitochondrial genomes and *clpC* gene for phylogenetic analysis used in this study | | | | | | |
| **GenBank accession no.** | | | | | **Haemosporidian Parasites** | **Vertebrate host** |
| **mtDNA** | ***cytb*** | ***coxI*** | ***coxIII*** | ***Clpc*** |  |  |
| OL999536 |  |  |  | KU133764 | *Plasmodium* sp. | *Odocoileus virginianus* |
|  | LC715200 | LC715187 | N/A | LC715197 | *Polychromophilus melaniphelus* | *Miniopterus fuliginosus* |
|  | LC668429 | LC715190 | N/A | LC715201 | *Polychromophilus melaniphelus* | *Miniopterus fuliginosus* |
|  | LC668430 | LC715191 | N/A | LC715202 | *Polychromophilus melaniphelus* | *Miniopterus fuliginosus* |
|  | LC668432 | LC715195 | N/A | LC715203 | *Polychromophilus murinus* | *Myotis macrodactylus* |
|  | LC668433 | LC715196 | N/A | LC715196 | *Polychromophilus murinus* | *Myotis macrodactylus* |
|  |  |  |  | KT750741 | *Polychromophilus* sp. | *Miniopterus natalensis* |
|  |  |  |  | MT750315 | *Polychromophilus* sp. | *Scotophilus kuhlii* |
